# Antioxidant Capacity of Rigenase^®^, a Specific Aqueous Extract of *Triticum vulgare*

**DOI:** 10.3390/antiox7050067

**Published:** 2018-05-17

**Authors:** Immacolata Antonucci, Gabriella Fiorentino, Patrizia Contursi, Massimiliano Minale, Rodolfo Riccio, Salvatore Riccio, Danila Limauro

**Affiliations:** 1 Dipartimento di Biologia, Università degli Studi di Napoli Federico II, Complesso Universitario Monte S. Angelo, 80126 Naples, Italy; immacolata.antonucci@unina.it (I.A.); fiogabri@unina.it (G.F.); contursi@unina.it (P.C.); 2 Farmaceutici Damor S.p.A, Via E. Scaglione 27, 80145 Naples, Italy; dirmed@farmadamor.it (M.M.); rodolforiccio@farmadamor.it (R.R.); salvatorericcio@farmadamor.it (S.R.)

**Keywords:** *Triticum vulgare*, antioxidant, reactive species of oxygen

## Abstract

Reactive species of oxygen (ROS), responsible for oxidative stress, accumulate in various tissues damaged by burns, decubitus ulcers, and vascular lesions. Antioxidants play an important and well-documented role in healing of chronic and acute wounds. Rigenase^®^, a specific extract of *Triticum vulgare* manufactured by Farmaceutici Damor, is employed in products used for the regeneration of tissue injuries. In this work, we show that Rigenase^®^ exhibits a scavenging effect toward free radicals, thus pointing to its relevant antioxidant activity.

## 1. Introduction

Reactive oxygen species (ROS), especially superoxide radicals (•O_2_^−^), hydrogen peroxide (H_2_O_2_), and hydroxyl radicals (•OH), are produced both during aerobic metabolism, as well as in response to external stimuli, or they are generated by environmental pollution [1]. The oxidative stress usually results from either excessive ROS generation, or impaired endogenous antioxidants or a combination of these events. Indeed, ROS high concentrations damage the main biological macromolecules, leading to proteins oxidation, lipid peroxidation, DNA base modifications, and strand breaks [2]. Therefore, ROS are implicated in the development and progression of cancer [3], neurodegenerative disorders such as Alzheimer’s and Parkinson’s diseases [4], as well as in inflammation [5], aging, and atherosclerosis [6]. Enzymatic and non-enzymatic antioxidant molecules are produced to protect the organisms from oxidative stress. Non-enzymatic antioxidants from natural sources are of great interest because they can be used to isolate phytochemicals with health benefits [7], in the prevention and/or curing of oxidative stress-related diseases [8].

*Triticum vulgare*, belonging to the family of Graminaceae, is the selected source used by Farmaceutici Damor to prepare Rigenase^®^, a specific aqueous extract from this plant. This is utilized to produce pharmaceutics, which are commonly used for the treatment of decubitus ulcers, burn lesions, scarring, and sores. All these injuries are associated with increased free radical production which might delay the wound healing process [9].

The role of antioxidants as molecules involved in skin regeneration and recovery of burn lesions is well documented [10]. In this work, we show that Rigenase^®^ exhibits antioxidant capacity, highlighting the functional versatility of this specific extract, documented both by tissue-repairing activity [5], and recently by moisturizing action (paper submitted).

## 2. Materials and Methods

### 2.1. Chemicals

Folin–Ciocalteu (F–C) reagent, gallic acid, 2,2-diphenyl-1-picrylhydrazyl (DPPH radical), 2,2′-azobis(2-amidinopropane) dihydrochloride (AAPH), and ascorbic acid were purchased from Sigma-Aldrich (St. Louis, MO, USA). OxiSelect™ Oxygen Radical Antioxidant Capacity (ORAC) Activity Assay kit was purchased from Cell Biolabs, Inc. (San Diego, CA, USA). Methanol was purchased from Romil Ltd. (Cambridge, UK). Sheep erythrocytes solution (100%) was purchased from Innovative Research Inc. (Peary Ct, Novi, MI, USA). All chemicals and reagents used were of analytical grade.

### 2.2. Plant Material

*T. vulgare* was grown under controlled conditions in the Farmaceutici Damor laboratory, Naples, Italy; the voucher specimen is DF/237/2014, and it is deposited in the herbarium of the Medical Botany Chain of University of Salerno, Italy. The commercially available seeds ware purchase from Consorzio Agrario Lombardo Veneto (San Giorgio sul Panaro, Modena, Italy).

In this study, RS1601, was a batch derived from a specific aqueous extract of *T. vulgare*, used to prepare Rigenase^®^ provided by Farmaceutici Damor. The obtained *T. vulgare* extract was sterilized through 0.2 μm membrane filter and stored at 4 °C.

### 2.3. Folin–Ciocalteu (F–C) Assay

The content in phenol/phenol-like and/or antioxidant compounds was determined with the F–C assay adapted to a 96-well microplate, as described [11,12]. A_765 nm_ was measured using a Synergy H4 microplate reader (BioTeK, Winooski, VT, USA) and compared against a standard curve of gallic acid; results were reported as mg of gallic acid equivalents (GAE) per liter, and were the average of three independent experiments ± Standard deviation (SD) each made in triplicate.

### 2.4. ORAC Assay

The ORAC assay was carried out using the OxiSelect™ Oxygen Radical Antioxidant Capacity (ORAC) Activity Assay kit, according to the manufacturer’s instruction. ORAC values were expressed as μmol Trolox Equivalents (TE) per gram of sample.

### 2.5. DPPH Assay

α,α-Diphenyl-β-picrylhydrazyl radical (DPPH) scavenging activity assay was determined as described [13]. Gallic acid was used as the reference compound. DPPH· inhibition percentage was calculated using the following formula:

%inhibition = [(A_0_ − A_t_)/A_0_ × 100]

where A_0_ and A_t_ were the absorbance values of the control (DPPH solution, without extract) and of the extract, respectively. Reported results were the average of three independent ± SD experiments each made in triplicate.

### 2.6. Sheep Erythrocytes Hemolysis Assay

Sheep erythrocytes hemolysis assay was performed using the following protocol: sheep erythrocytes were washed three times with 5 volume of phosphate-buffered saline (PBS). Sheep erythrocytes at a final concentration of 5% were incubated with 4.4 mg/mL of Rigenase^®^ or ascorbic acid, used as reference compound [14], for 30 min at 37 °C. Then 200 mM AAPH was added to the mixtures and incubated at 37 °C; erythrocytes hemolysis was monitored every 20 min in the supernatants by spectrophotometric readings at A_524 nm_. Hemolysis percentage was calculated by the formula: % hemolysis = [(A_sample_/A_positive control_) × 100]; whereas the positive control consisted of a mixture of erythrocyte suspension and water (to obtain 100% hemolysis) [14]. Reported results, expressed as mean ± SD, were the average of three independent experiments each made in triplicate. Statistical comparison between the reference compound and Rigenase^®^ samples was performed using the using the one-way Analysis of Variance (ANOVA). A *p*-value < 0.05 was considered statistically significant.

## 3. Results and Discussion

### 3.1. Rigenase^®^ Antioxidant Capacity

Rigenase^®^, a specific aqueous extract of *T. vulgare*, has been tested for its antioxidant capacity by F–C method, ORAC, and DPPH assay.

The former is a simple method to describe antioxidant capacity based on the single electron transfer from various substrates, such as phenols or other antioxidant compounds [12], to the complexed Mo(VI) present in F–C reagent. Our results showed that Rigenase^®^ has an antioxidant compounds content of 61.2 ± 3 mg GAE/L, a value that is lower than that of fruit juices, but comparable to that of goat and/or cow milk, and elderflower beverages [15,16,17].

**Figure 1:**
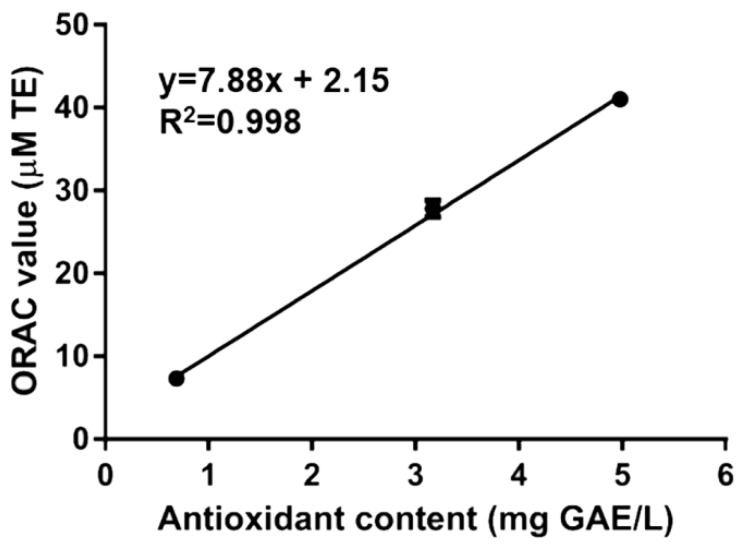
Linear correlation of antioxidant content vs. Oxygen Radical Antioxidant Capacity (ORAC) value of Rigenase^®^. Correlation analysis has been carried out by using GraphPad Prism 6.01 software (La Jolla, CA 92037, USA).

To confirm the presence of antioxidant molecules, Rigenase^®^ has also been analyzed by ORAC assay. This method is based on the oxidation of the fluorescein by peroxyl radicals (ROO), with a hydrogen atom transfer (HAT) process. The ORAC value (Table 1), measured as Trolox equivalents, was lower than Maqui fruit [18], but higher then roasted Yak-kong [19] or *Koji* mold (*Aspergillus* sp.) [20]. The correlation between the content of antioxidant compounds measured by F–C and ORAC values of Rigenase^®^ was determined by using regression analysis (Figure 1). The determined correlation coefficient (*R*^2^) was 0.998, suggesting that redox activity of the extract could be provided by phenol like/antioxidant compounds.

To go insight into the antioxidant capacity, the DPPH assay was also performed on Rigenase^®^. The DPPH radical is largely used to evaluate free radical scavenging activity because of the easiness of the reaction. As showed in Table 2, the DPPH radical scavenging activity measured on Rigenase^®^, was comparable to that determined for different vegetable and fruit extracts [7,13,19].

### 3.2. Inhibitory Effect of Rigenase^®^ on Sheep Erythrocyte Hemolysis

The antioxidant capacity of Rigenase^®^ has also been assessed through oxidative hemolysis inhibition assay, which is based on inhibition of free radical-induced membrane damage in sheep erythrocytes by antioxidants. The results obtained reflect biologically relevant radical scavenging activity, and microlocalization of antioxidants.

**Figure 2:**
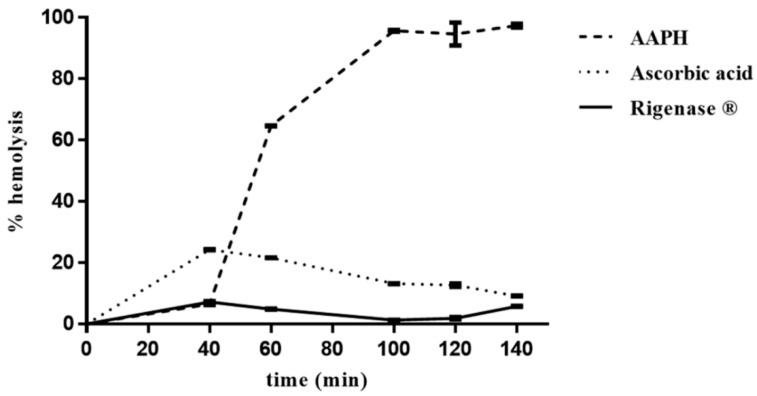
Inhibition of AAPH-induced erythrocyte hemolysis by Rigenase^®^. Erythrocytes suspension was incubated with AAPH at 37 °C in the absence (----) and in the presence of Rigenase^®^ (^___^) or ascorbic acid (····). Each value, from which the spontaneous hemolysis was subtracted, is the mean ± SD of three separate experiments.

The erythrocytes hemolysis was monitored spectrophotometrically (A_524 nm_), every 20 min, from the addition of AAPH, which induces generation of free radicals. In the absence of the extract, the percentage of erythrocytes hemolysis was about 6%, 40 min after onset of oxidative stress, rising up to 100% after 100 min (Figure 2). Conversely, in the presence of the extract, the degree of hemolysis was constant (~6%) up to 140 min, thus pointing to its radical scavenging activity. Interestingly, this result shows that Rigenase^®^ performs better than ascorbic acid (Figure 2).

## 4. Conclusions

A specific aqueous extract named Rigenase^®^ was isolated by Farmaceutici Damor from the natural source of *T. vulgare*. The antioxidant capacity of Rigenase^®^ was clearly demonstrated by different assays. Interestingly, the extract shows a stronger inhibition of AAPH-induced erythrocytes hemolysis than ascorbic acid, highlighting its relevant radical scavenging activity. All together, our results represent the first step towards the characterization of the antioxidant capacity of Rigenase^®^ that could be exploited in the treatment of burns and injuries characterized by high concentration of the free radicals.

## Figures and Tables

**Table 1:** Comparative analysis of antioxidant capacity of Rigenase^®^ by Oxygen Radical Antioxidant Capacity (ORAC) activity assay.

Sample	μmol Trolox Equivalents (TE)/g	References
Rigenase^®^	74 ± 16	This work
Roasted Yak-kong (black beans)	6.89	[19]
Maqui fruits	194–241	[18]
*Koji* mold (*Aspergillus* sp.)	38.3	[20]

**Table 2:** Comparative analysis of antioxidant capacity of Rigenase^®^ by DPPH assay.

Sample	% DPPH Inhibition	References
Rigenase^®^ (6 mg/mL)	89 ± 6	This work
*Stevia rebaudiana* leaf extract (0.2 mg/mL)	69	[7]
*Ulva intestinalis* Linnaeus (2 mg/mL)	45.8	[13]
Roasted Yak-kong (2.2 mg/mL)	50	[19]
